# Supplementary material for: EEG signature of breaks in embodiment in VR
Source: PLoS One. 2023 May 11;18(5):e0282967. doi: 10.1371/journal.pone.0282967 (PMC10174550; doi:10.1371/journal.pone.0282967)
Supplement: Attachment — Submitted filename: Response to Reviewers.pdf [file pone.0282967.s001.pdf]

Dear Editor and Reviewers,

Thank you for reviewing our manuscript entitled "EEG signature of breaks in embodiment in VR" and for providing constructive feedback. We greatly appreciate your comments and have studied them carefully. In the following, we address the reviewers' comments point by point and explain how the paper has been revised accordingly.

For your convenience, reviewers' comments are in black, our responses are in blue, and revised text in the manuscript is in red. The answers to the reviewers can be found at the following pages of the current document:

- Editor: page 2- 3
- Reviewer 1: page 4 - 5
- Reviewer 2: page 6 - 9

Thank you again for your time in reviewing our manuscript.

Yours sincerely,

Authors of the manuscript

Thibault Porssut, Fumiaki Iwane, Ricardo Chavarriaga, Olaf Blanke, José del R. Millán, Ronan Boulic and Bruno Herbelin

Editor: When submitting your revision, we need you to address these additional requirements.

1. Please ensure that your manuscript meets PLOS ONE's style requirements, including those for file naming. The PLOS ONE style templates can be found at

https://journals.plos.org/plosone/s/file?id=wjVg/PLOSOne_formatting_sample_main_body.pdf and
https://journals.plos.org/plosone/s/file?id=ba62/PLOSOne_formatting_sample_title_authors_affiliations.pdf

We formatted our manuscript following PLOS one style as requested.

2. Please change "female" or "male" to "woman" or "man" as appropriate, when used as a noun (see for instance https://apastyle.apa.org/style-grammar-guidelines/bias-free-language/gender).

We corrected our manuscript as suggested.

3. Please update your submission to use the PLOS LaTeX template. The template and more information on our requirements for LaTeX submissions can be found at http://journals.plos.org/plosone/s/latex.

We used Latex template to convert our manuscript as suggested.

4. Financial disclosure:

This work was supported by the Swiss National Science Foundation (project 'Immersive Embodied Interactions', 200020.178790), the Hasler Foundation (16070), Switzerland, and by the Swiss National Center of Competence in Research in Robotics (NCCR). The funders had no role in study design, data collection and analysis, decision to publish, or preparation of the manuscript.

5. We note that you have stated that you will provide repository information for your data at acceptance. Should your manuscript be accepted for publication, we will hold it until you provide the relevant accession numbers or DOIs necessary to access your data. If you wish to make changes to your Data Availability statement, please describe these changes in your

cover letter and we will update your Data Availability statement to reflect the information you provide.

The entire anonymized dataset is available on the zenodo.org platform under the DOI 10.5281/zenodo.7524989. The following link allows accessing it online:

https://doi.org/10.5281/zenodo.7524989

6. Please review your reference list to ensure that it is complete and correct. If you have cited papers that have been retracted, please include the rationale for doing so in the manuscript text, or remove these references and replace them with relevant current references. Any changes to the reference list should be mentioned in the rebuttal letter that accompanies your revised manuscript. If you need to cite a retracted article, indicate the article's retracted status in the References list and also include a citation and full reference for the retraction notice.

We confirm that the reference list is complete and correct. All the added references have been indicated in the reviewed manuscript with track changes.

Reviewer #1: The authors present an interesting study on embodiment in virtual reality studying the neural mechanisms during disruption of ownership illusions, otherwise known as breaks in embodiment, through electrical neuroimaging data acquisition. In line with previous work, the authors report error-related potentials when visuo-proprioceptive correlations are not present (disruption of agency) and that the amplitude of the errors is modulated under the different experimental conditions of manipulation of the sense of embodiment.

In general, the study is well-structured and presented regarding the introduction, methodology, data analysis, and discussion. The authors report adequately on the background literature to later support their hypothesis and argue their findings. I find the study of great importance for researchers in the field and for advancing knowledge.

I do have some questions on a couple of points that were not clear not.

1. First, regarding the subjective reports, why was only one question included in the assessment referring to the extent participants felt that the virtual body was theirs ('I felt as if the virtual body was my body'), whereas in previous studies additional questions also related to agency are usually included? See examples Peck TC and Gonzalez-Franco M (2021) Avatar Embodiment. A Standardized Questionnaire or Roth D and Latoschik ME (2020) among others.

Thank you for expressing this comment, which was indeed a design choice that might not appear obvious or justified. We added the following sentences to the manuscript;

In page 5, lines 119: "Because the experimental design requests a large number of repetitions, we wanted to minimize the number of questionnaire items. We decided not to ask for a subjective rating of the sense of agency as it would only have been useful to confirm that participants were aware of our manipulation, and we had no reason to hypothesize that it would not be the case[17]. "

2. Second, though the number of participants is relatively low the within-groups design allows for good comparisons. Nonetheless, I did not see in the text if the different experimental conditions were counterbalanced and now these were presented to participants over the different blocks and trials.

Thank you for noticing this missing information. We added the following sentences to the manuscript.

In page 5, line 104: "In each block, we ensured the ratio of each condition and the trials were counterbalanced to compensate for the order effect. Each participant performed 120 trials of *Non-Embodied/No Disruption and Embodied/No Disruption*, 60 trials of *Non-*

Embodied/Disruption and *Embodied/Disruption.* The number of trials was not available to participants. "

3. Last, the figures in the submitted manuscript are of low quality and need to be upgraded, and a video demonstration of the experimental setup would help readers better understand the scenario and discuss manipulations.

Sorry that the figure quality was lost in the pre-print to PLOS-ONE process: we will ensure that high quality figures are published. Please find a link to a video of the experiment: https://youtu.be/Pg1cqpwDas8

Reviewer #2: The article deals with an interesting topic, namely the breaks in the embodiment by providing behavioral and electroencephalography original data. Overall, the results are intelligible and well presented, including the clear mention of the limits (e.g. absence of clean error-related potentials analysis during motor actions), which are then manifestly available to the reader. I provide below just a few point-by-point suggestions and comments that I hope will contribute to improving the quality of the authors' work.

1. Given the relevance of the manipulation, I suggest the authors clearly state and repeat in the initial part of the method section the usefulness of each of the two steps included in the experimental phase (i.e. embodiment to create the embodiment and disruption to manipulate the feeling eventually created in the previous phase). Personally, I do not find the term 'monitoring' entirely appropriate and would suggest finding an alternative formulation

Thanks for your suggestion on how to improve the explanation of our experimental approach. We added the following sentence in the beginning of the paragraph explaining the two steps:

In page 3, line 77: "The succession of the two steps is key for our observations; first we induce a sense of embodiment (or not) for the avatar and second, we disrupt this sense of embodiment (or not) while participants are passively monitoring the virtual arm to prevent any eye movement or motion artifacts in the EEG signal)."

Regarding the choice of the term 'monitoring', it is based on the literature on 'error monitoring' and 'performance monitoring'; while we understand that it might not be ideal, we couldn't converge to a better alternative (e.g. 'observation' or 'waiting' are too passive, 'task' is incorrect as the task includes two steps)

2. Wisely the authors include different time intervals within the experimental phase, however, this experimental choice is not fully explained. I suggest adding an explanation of the choice

As pointed out, we employed the time intervals of 0.9, 1.5 and 2.1 s. The choice of these duration was to prevent participants from knowing the exact moment when the next step starts. We added the following sentence to the manuscript to describe our choice of the time intervals.

In page 4, line 87: "These randomized time intervals were to prevent participants from knowing the exact starting time of the next step."

3. In principle, the same task as in step 1 could be repeated to disrupt the embodiment. Can the authors specify the rationale behind the choice of changing the task from a visuo-motor

to a visuo-proprioceptive (e.g. avoiding artifacts?) and discuss the possible consequences or whether or not they believe there may be any?

We modified the tasks between step 1 and step 2 for two reasons; to remove active motor actions and to temporally align when participants perceive Break-in-Embodiment.

In page 4, line 93: "We employed different tasks between step 1 and step 2 to remove active motor action of their wrist and to temporally align when participants perceive Break-in-Embodiment."

4. The authors only investigate embodiment at the end of the entire experimental procedure (steps 1 and 2). Why was embodiment not also investigated after step 1 alone, to verify (at least on a behavioral level) that the manipulation was successful?

The experiment was already long (2h in total). So we had to restrict the number of questions. Moreover, thanks to the answers during the Embodied*/No Disruption* condition where participants were embodied with no disruption we could ensure that participants were embodied during the first step. On the contrary, thanks to the answers in the *Non-Embodied/No Disruption* condition, we could ensure that participants were not embodied.

Concerning the EEG measurement, we did not include this time window for the EEG analysis as participants moved their wrist at that time and their movements were different (speed, onset and direction of movement). Any conclusion we could have made regarding humans' cognitive function on Break-in-Embodiment from this time window of EEG may be contaminated by differential movement patterns.

In page 4, line 95: "Due to time constraints, no questions were asked at the end of step 1. We ensured that participants were embodied or not during this first step thanks to the answers during *Embodied/No Disruption and Non-Embodied/No Disruption* condition."

In page 5, line 147: "We restricted the subsequent EEG analysis to the monitoring step as participants performed motor actions in the induction step, which may be a confounding factor when evaluating humans' cognitive process."

5. Does the duration of the embodiment phase (time interval from active induction to monitoring phase) have an impact on the results? are there differences related to the different time intervals involved?

To answer this question, we performed additional analysis to see the effect of time intervals between the two steps. As depicted in the figure below, we did not observe any difference linked to the different time intervals. To report this result, we added the following sentence in the manuscript.

In page 8, line 191: "We did not observe any effect in EEG signals associated with different interval durations."

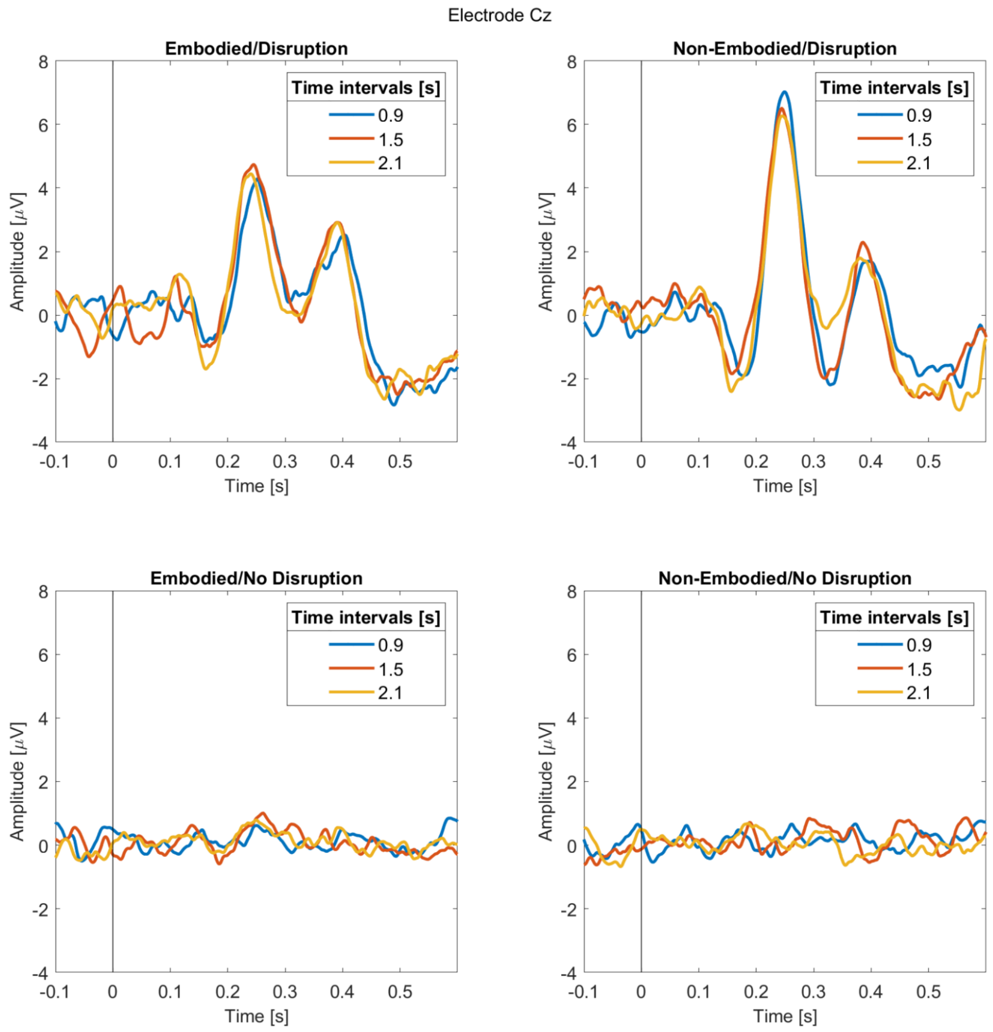

6. In general, I find the figures helpful and well thought out. In figure 1, adding labels to explain the different components of the setup could improve the quality of the picture

Thank you for this comment. We have updated figure 1 with the suggested labels.

7. Recent studies have demonstrated the relevance of the virtual reality environment (coupled with sensory stimulation) in the field of rehabilitation (Risso Preatoni et al. 2022: https://doi.org/10.1016/j.isci.2022.104129 ; Risso & Valle https://doi.org/10.1007/s40141-022-00350-x ; Serino et al. 2022 https://www.cairn.info/revue-de-neuropsychologie-2022-1-page-15.html ; Rognini et al 2019 http://dx.doi.org/10.1136/jnnp-2018-318570). It would be interesting to discuss the potential impact of these findings on the clinical setting.

We thank the reviewer for suggesting this interesting clinical application of our work; a sentence was added as an outlook for a clinical application, with two references.

In page 10, line 287: "This approach could also be beneficial for the clinical evaluation of embodiment of prosthetic limbs, with benefits for reducing phantom limb distortions and pain in hand[45] or leg[46] amputee."

Typos:

- Section 1 introduction : […]Errorrelated Potentials (ErrPs) induced by disruptions in VR already […]

- Section 2.2. Subjective rating: […] withingroup comparison using the Benjamini-Hochberg[…]

Thank you for pointing them out. We corrected these typos in the updated manuscript.
